# Supplementary material for: MetaRibo-Seq measures translation in microbiomes
Source: Nat Commun. 2020 Jun 29;11:3268. doi: 10.1038/s41467-020-17081-z (PMC7324362; doi:10.1038/s41467-020-17081-z)
Supplement: Supplementary file 10 — Supplementary Data 7 [file 41467_2020_17081_MOESM10_ESM.zip › File2/Confidence_High_Taxonomy/333074_out.krona.html]

Javascript must be enabled to view this page.

members
magnitude
magnitudeUnassigned
count
unassigned
taxon
rank

333074\_out

154

154

SRS016381\_contig\_number\_contig-100\_5384.5385SRS018541\_contig\_number\_contig-100\_1052.95312SRS019381\_contig\_number\_contig-100\_10732.85577SRS098827\_contig\_number\_contig-100\_1232.74937SRS1055076\_contig\_number\_contig-100\_3375.102213SRS147766\_contig\_number\_contig-100\_3688.145092SRS148091\_contig\_number\_14905SRS149879\_contig\_number\_28245SRS893366\_contig\_number\_22726SRS971275\_contig\_number\_contig-100\_4923.253824
superkingdom
10
2

9
1239

SRS012969\_contig\_number\_contig-100\_1362.247306SRS019808\_contig\_number\_contig-100\_3387.125068SRS042966\_contig\_number\_contig-100\_1870.129674SRS064276\_contig\_number\_contig-100\_891.231965SRS077849\_contig\_number\_17660SRS098655\_contig\_number\_14235SRS1041137\_contig\_number\_contig-100\_3867.179288SRS147557\_contig\_number\_contig-100\_2376.184566SRS148196\_contig\_number\_contig-100\_6666.119091
phylum
143

134
class
186801

186802
32
order

SRS011134\_contig\_number\_contig-100\_1391.294012SRS011239\_contig\_number\_26756SRS013215\_contig\_number\_contig-100\_1771.19405SRS013476\_contig\_number\_contig-100\_1664.217872SRS013687\_contig\_number\_contig-100\_2152.194807SRS013965\_contig\_number\_40359SRS014287\_contig\_number\_contig-100\_2429.118831SRS016056\_contig\_number\_2432SRS017433\_contig\_number\_11736SRS018656\_contig\_number\_12307SRS019910\_contig\_number\_3772SRS024132\_contig\_number\_contig-100\_1572.284560SRS043001\_contig\_number\_contig-100\_5578.74255SRS048981\_contig\_number\_8739SRS049402\_contig\_number\_contig-100\_2509.119231SRS064757\_contig\_number\_24900SRS075398\_contig\_number\_contig-100\_877.29545SRS077552\_contig\_number\_contig-100\_1347.176749SRS077641\_contig\_number\_contig-100\_2681.123912SRS077730\_contig\_number\_contig-100\_1928.113389SRS1041031\_contig\_number\_28690SRS1041095\_contig\_number\_contig-100\_982.40268SRS143085\_contig\_number\_contig-100\_1246.50299SRS143780\_contig\_number\_contig-100\_5019.5019SRS143780\_contig\_number\_contig-100\_9760.153202SRS143991\_contig\_number\_contig-100\_2221.177537SRS144714\_contig\_number\_contig-100\_2920.175302SRS146888\_contig\_number\_contig-100\_7984.80236SRS147022\_contig\_number\_14135SRS147919\_contig\_number\_contig-100\_8029.94020SRS893230\_contig\_number\_contig-100\_1416.82219SRS893380\_contig\_number\_contig-100\_324.34265
134


SRS016954\_contig\_number\_26253SRS051882\_contig\_number\_14173SRS057717\_contig\_number\_contig-100\_825.57188SRS140645\_contig\_number\_contig-100\_3823.24193
family
46
4
186803

22
33042
genus

1
species
410072

SRS140492\_contig\_number\_34661


SRS011405\_contig\_number\_5822SRS017191\_contig\_number\_contig-100\_10417.87266SRS018313\_contig\_number\_contig-100\_1715.87869SRS019445\_contig\_number\_31470SRS020328\_contig\_number\_38563SRS024331\_contig\_number\_13659SRS043701\_contig\_number\_2339SRS045244\_contig\_number\_contig-100\_2349.69013SRS049959\_contig\_number\_38762SRS053214\_contig\_number\_7123SRS053356\_contig\_number\_44347SRS057480\_contig\_number\_8514SRS075078\_contig\_number\_33384SRS075716\_contig\_number\_contig-100\_558.46771SRS077294\_contig\_number\_19413SRS077454\_contig\_number\_19465SRS077502\_contig\_number\_contig-100\_5384.90636SRS097920\_contig\_number\_contig-100\_1439.194219SRS101433\_contig\_number\_contig-100\_2642.112267SRS142599\_contig\_number\_23144SRS143148\_contig\_number\_contig-100\_1588.160083
1896998
species
21

genus
1506553
1

1

SRS014235\_contig\_number\_contig-100\_1476.267901
1531
species

4
genus
1506577

29361
species

SRS017103\_contig\_number\_43844SRS046369\_contig\_number\_contig-100\_1034.95254SRS046712\_contig\_number\_contig-100\_1460.30148SRS144297\_contig\_number\_14933
4

1
genus
841


SRS1041142\_contig\_number\_7757
512314
species
1

1
189330
genus

1

SRS017521\_contig\_number\_contig-100\_2774.269592
39486
species

7
572511
genus

2
species
2292963

SRS077231\_contig\_number\_26667SRS1041129\_contig\_number\_13797

1

SRS097889\_contig\_number\_139
33038
species


SRS014948\_contig\_number\_22251
species
2292967
1

2

SRS053335\_contig\_number\_contig-100\_1745.113353SRS147727\_contig\_number\_contig-100\_246.247
species
2292970

1
species
33035

SRS893288\_contig\_number\_4203

2

SRS013951\_contig\_number\_contig-100\_1005.195001SRS019161\_contig\_number\_42504
2302974
species


SRS076876\_contig\_number\_1361SRS1041112\_contig\_number\_contig-100\_1708.49324SRS104311\_contig\_number\_2399
658655
species
3

genus
28050
1

1
species
446043

SRS144362\_contig\_number\_contig-100\_1204.260854

31979
1
44
family

SRS017849\_contig\_number\_357

2
1485

SRS064557\_contig\_number\_contig-100\_2413.128772SRS1055034\_contig\_number\_contig-100\_942.79005
genus
40

1
species
2293024

SRS055017\_contig\_number\_30903


SRS075021\_contig\_number\_5685
59620
species
1


SRS053398\_contig\_number\_23924
species
2293041
1


SRS012902\_contig\_number\_contig-100\_1023.15538SRS015190\_contig\_number\_contig-100\_1329.99243SRS015578\_contig\_number\_1825SRS015579\_contig\_number\_contig-100\_565.155076SRS016267\_contig\_number\_13256SRS018936\_contig\_number\_contig-100\_600.92409SRS022137\_contig\_number\_22422SRS022713\_contig\_number\_5950SRS023715\_contig\_number\_contig-100\_1974.177749SRS023829\_contig\_number\_867SRS042628\_contig\_number\_37262SRS048060\_contig\_number\_contig-100\_742.84304SRS051610\_contig\_number\_contig-100\_6422.44515SRS055982\_contig\_number\_contig-100\_1860.121231SRS063370\_contig\_number\_contig-100\_764.88629SRS077392\_contig\_number\_8122SRS1041130\_contig\_number\_6017SRS1041147\_contig\_number\_contig-100\_1048.32498SRS1041164\_contig\_number\_contig-100\_959.45474SRS1055043\_contig\_number\_3070SRS1055049\_contig\_number\_6335SRS1055099\_contig\_number\_contig-100\_1313.82551SRS143342\_contig\_number\_20317SRS144183\_contig\_number\_contig-100\_2796.136348SRS144603\_contig\_number\_contig-100\_1730.90575SRS144753\_contig\_number\_contig-100\_655.88016SRS147653\_contig\_number\_contig-100\_808.46646SRS148970\_contig\_number\_contig-100\_1020.40542SRS893292\_contig\_number\_contig-100\_4606.53029SRS893295\_contig\_number\_contig-100\_5603.65092SRS893300\_contig\_number\_7218SRS893383\_contig\_number\_33204
1720194
species
32

2292206
species

SRS015369\_contig\_number\_7679SRS103987\_contig\_number\_37336SRS105153\_contig\_number\_6921
3

2
420345
genus

2

SRS043411\_contig\_number\_15298SRS142980\_contig\_number\_1827
341220
species

1
genus
580596

1
2292197
species

SRS049773\_contig\_number\_contig-100\_2474.212515

9
family
541000


SRS065176\_contig\_number\_11649
1535
species
1

genus
1263
7

2

SRS015663\_contig\_number\_7480SRS051031\_contig\_number\_28133
46228
species

2293232
species

SRS016753\_contig\_number\_contig-100\_2344.57634SRS018623\_contig\_number\_13384SRS1041140\_contig\_number\_22037SRS1055038\_contig\_number\_contig-100\_2059.2060SRS1055067\_contig\_number\_5325
5

1
genus
292632

1
214851
species

SRS104912\_contig\_number\_2069

1
186806
family

1
genus
1730

142586
species

SRS148159\_contig\_number\_57034
1

1
216572
family

genus
459786
1

1
1945593
species

SRS065397\_contig\_number\_3148

species
1897049

SRS023914\_contig\_number\_20668
1

976
phylum
1

200643
class
1

1
171549
order

family
815
1

816
genus

SRS148721\_contig\_number\_contig-100\_3631.316267
1
